# A power amplification dyad in seahorses

**DOI:** 10.1098/rspb.2023.0520

**Published:** 2023-04-12

**Authors:** Corrine Avidan, Steven W. Day, Roi Holzman

**Affiliations:** ^1^ Department of Ecology, Evolution and Organismal Biology, Brown University, Providence, RI 02912, USA; ^2^ School of Zoology, Faculty of Life Sciences, Tel Aviv University, Tel Aviv 69978, Israel; ^3^ The Inter-University Institute for Marine Sciences, POB 469, Eilat 88103, Israel; ^4^ Rochester Institute of Technology, Rochester, NY 14623, USA

**Keywords:** LaMSA, elastic energy storage, suction feeding, syngnathidae, muscle power, seahorse

## Abstract

Throughout evolution, organisms repeatedly developed elastic elements to power explosive body motions, overcoming ubiquitous limits on the power capacity of fast-contracting muscles. Seahorses evolved such a latch-mediated spring-actuated (LaMSA) mechanism; however, it is unclear how this mechanism powers the two complementary functions necessary for feeding: rapidly swinging the head towards the prey, and sucking water into the mouth to entrain it. Here, we combine flow visualization and hydrodynamic modelling to estimate the net power required for accelerating the suction feeding flows in 13 fish species. We show that the mass-specific power of suction feeding in seahorses is approximately three times higher than the maximum recorded from any vertebrate muscle, resulting in suction flows that are approximately eight times faster than similar-sized fishes. Using material testing, we reveal that the rapid contraction of the sternohyoideus tendons can release approximately 72% of the power needed to accelerate the water into the mouth. We conclude that the LaMSA system in seahorses is powered by two elastic elements, the sternohyoideus and epaxial tendons. These elements jointly actuate the coordinated acceleration of the head and the fluid in front of the mouth. These findings extend the known function, capacity and design of LaMSA systems.

## 1. Introduction

Organismal performance is often limited by muscle power, and this is especially true for fast, explosive motions. This limitation stems from an innate trade-off between muscle force and contraction speed, the product of which is power [1,2]. Latch-mediated spring-actuated (LaMSA) systems, in which slow forceful muscles are used to load an elastic element that is kept latched until it is allowed to abruptly release, have evolved in many organisms and have overcome muscle power limitations [3]. LaMSA systems enable some of the fastest animal activities, powering the movements of jumping legs in fleas [4,5], the shell-breaking appendages of mantis shrimps [6,7] and the closing jaws in trap-jaw ants [8]. Although variations of LaMSA systems have been described in dozens of invertebrates [9], variations in vertebrates are rare, but include the ballistic tongues of anurans and chameleons and the jumping legs in frogs [3,10,11].

**Figure 1.**
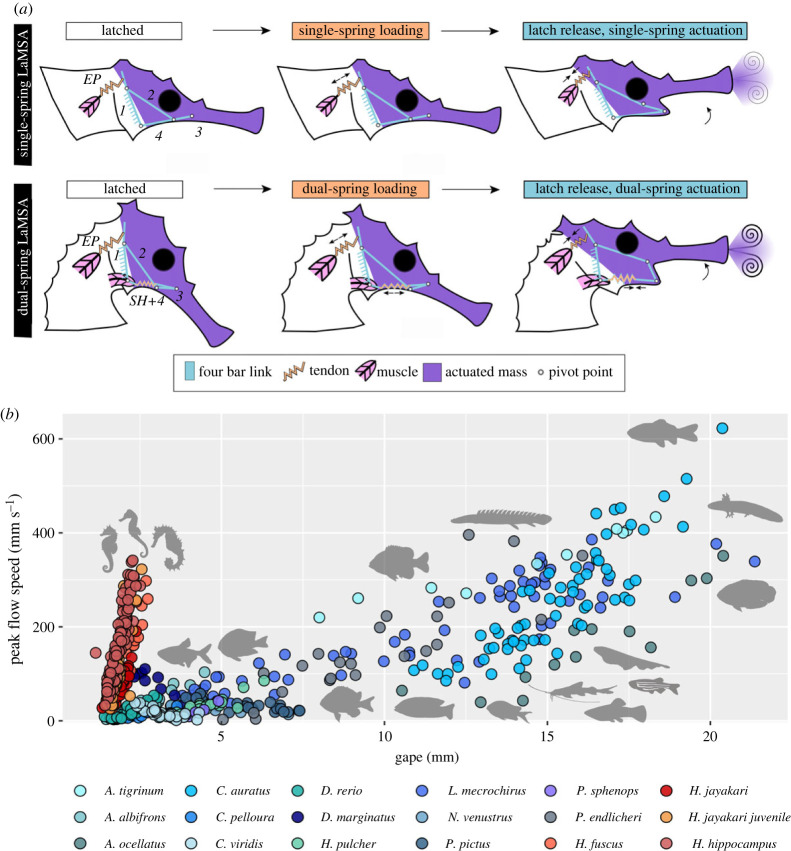
(*a*) Schematic illustrations of LaMSA systems in Syngnathiformes and the four-bar linkage system that transmits motion from the elastic tendons to lift the head and expand the buccal cavity. In single-LaMSA fishes such as the snipefishes (upper row), the epaxial tendons (EP) are the only elastic element that powers the system [14]. Power is transmitted through a rigid four-bar linkage to lift the head and depress the hyoid, thus powering both pivot feeding and probably suction feeding [14]. In the locked configuration, the tendons are loaded, and the head cannot be raised. When the system is triggered and the hyoid rotates downwards, the tendons recoil, simultaneously pulling levers 2 and 3. In seahorses (lower row), the four-bar linkage is modified to include the sternohyoideus muscle-tendon complex, and we hypothesize that this complex can store elastic energy within the four-bar loop [15] and accelerate the suction flows. Indeed, flow visualization using particle imaging velocimetry indicated that seahorses generate suction flows that are eight times stronger than expected based on their size ((*b*), [16]). Notation of LaMSA components follows [3,17]. The epaxial and sternohyoideus tendons are noted as EP and SH, respectively. See the electronic supplementary material, methods section ‘Morphology’ for details of the functional anatomy. Colours in (*b*) depict different species, with warm colours depicting species with an LaMSA system and cooler colours depicting species without this system. Silhouettes of the represented species are located at approximately peak flow speed positions (*y*-axis).

An additional rare vertebrate example is an LaMSA system that evolved within Syngnathiformes [3,12], the order containing the power-amplified seahorses, pipefishes and snipefishes. Fishes within these families capture prey using ‘pivot feeding’, a behaviour comprising an abrupt upward rotation of the head towards the prey. Pivot-feeding fishes can open their mouth and elevate their head within a few milliseconds, reaching angular speeds of 200 rad s^−1^. Head rotation is powered by the rapid recoil of a pair of epaxial tendons, which are pre-loaded by the epaxial muscles long before the fish is ready to strike [12,13]. Power is transmitted from the elastic elements to elevate the head and rotate the hyoid by a four-bar linkage system, consisting of four links (bones and tendons) that are connected in a loop by four joints (figure 1*a*; [12,14]). In systems featuring four rigid bars, the movement of any bar results in predictable, coordinated movements of the other three bars [15]. Such a four-bar systems is known to transmit power to elevate the head in LaMSA-powered snipefishes [3,14], to the smashing appendages in mantis shrimps [6,7,15,18], and transmit muscle power within other non-LaMSA muscle-skeletal systems [19–21]. In seahorses, a four-bar system is used to transmit power to elevate the head, but unlike the rigid four-bar system present in snipefishes [14], the lower bar in seahorses is not rigid (figure 1*a*; [22]). This bar is composed of a short bone (the urohyal) and a tendon-muscle complex (hereafter the sternohyoideus tendons; see the electronic supplementary material, section ‘*Morphology*’). *In vivo* recordings of action potentials from that muscle complex indicate that it contracts prior to the strike, suggesting that it probably loads the sternohyoideus tendons to potentially store elastic energy [22,23]. It is thought that the system is latched by contracting the adductor arcus palatini muscle that keeps the hyoid in a proximal (forward-pointing) position [14,24] and that muscle-actuated release and retroversion of the hyoid trigger head elevation [14,23,24]. Alternatively, it is possible that the system is latched by an over-centring trigger mechanism [3,25] as suggested for the closely related snipefishes [15].

The rapid motion of the elongated snout allows seahorses to specialize in feeding on evasive prey by bringing the mouth close to the prey before it can initiate an escape response [26]. However, bringing the mouth close to the prey is not sufficient for prey capture. To successfully engulf their prey, the fish must generate a flow of water external to their mouth that carries the prey inside. These ‘suction flows’ ubiquitously persist for a short period and are effective only over small spatial scales [27,28]. Therefore, to be effective in capturing evasive prey, suction flows must occur during head elevation. Previous analysis of the flow field generated by suction feeding non-LaMSA fishes and seahorses ([16,27]; electronic supplementary material, movies S2–S4, ‘*Particle image velocimetry*’ and table S1) revealed that seahorses generate suction flows that are 8x faster than expected based on their mouth diameter. The estimated slope (±s.e.) that represents the relationship between gape size and suction flow speed in seahorses was 202 ± 9.8, whereas that slope was 24.6 ± 0.9 for non-LaMSA fishes [16,27] (figure 1*b*; phylogenetically informed mixed-effect model; Bayes factor > 10^5^; see the electronic supplementary material, table S2 for model results and details on statistical model). Pivot-feeding strikes of seahorses were extremely fast, characterized by time to peak gape of 2.5 ± 0.18 ms and head rotation speeds of 200 rad s^−1^. In seahorses, suction flows peaked within 2.1 ± 0.011 ms, an order of magnitude faster than that of other actinopterygians (e.g. bluegill *Lepomis macrochirus*: 33 ± 4 ms [27]). However, it is still unclear whether these flows are augmented by elastic energy storage, and if so, what mechanics support the storage and actuation of this energy?

In this study, we used a large dataset of the spatial and temporal pattern of suction flows in 10 species of non-LaMSA fishes and three seahorse species to estimate the power needed to accelerate the suction flows into the mouth. We then compared these estimates to the muscle mass-specific power across LaMSA- and non-LaMSA species. Lastly, we combine material testing and a mathematical reconstruction of the hyoid and head movements to calculate the potential for elastic storage of the required suction flow power by the sternohyoideus tendons.

## 2. Results

### (a) Net suction power

**Figure 2.**
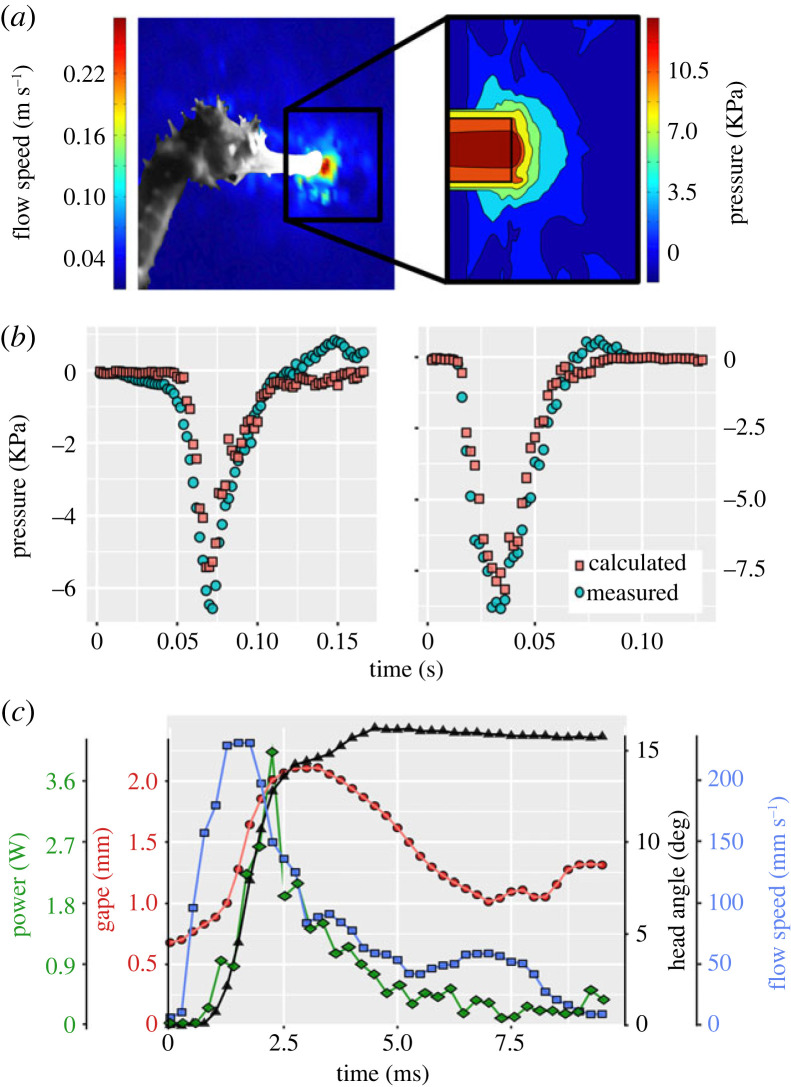
Experimental approach. (*a*) We used detailed flow fields from PIV experiments to estimate the pressure fields in front of the mouth (inset in (*a*); following [30,31]). The PIV-based estimation of suction pressure was validated for an independent dataset (*b*) where recordings of buccal pressure and external flow fields were taken simultaneously for largemouth bass (*Micropterus salmoides*; [32]; see the electronic supplementary material, ‘*Suction flow power*' and figure S3). The correspondence between measured and calculated pressure was very high (*R*^2^ = 0.92). An example of the timing of events during a seahorse strike is shown in (*c*), where the peak flow speed occurs first followed by peak power, then maximum gape opening and lastly peak head rotation. See the electronic supplementary material, figure S4 for kinematic profiles and mean timings for all Syngnathid species used. An image of the studied animal in (*a*) is superimposed on a false colour image, depicting the spatial distribution of suction flows at the time of peak flow speed (left panel). The black rectangle in the inset in (*a*) represents the snout, and the resulting pressure gradient is shown inside and outside of it.

**Figure 3.**
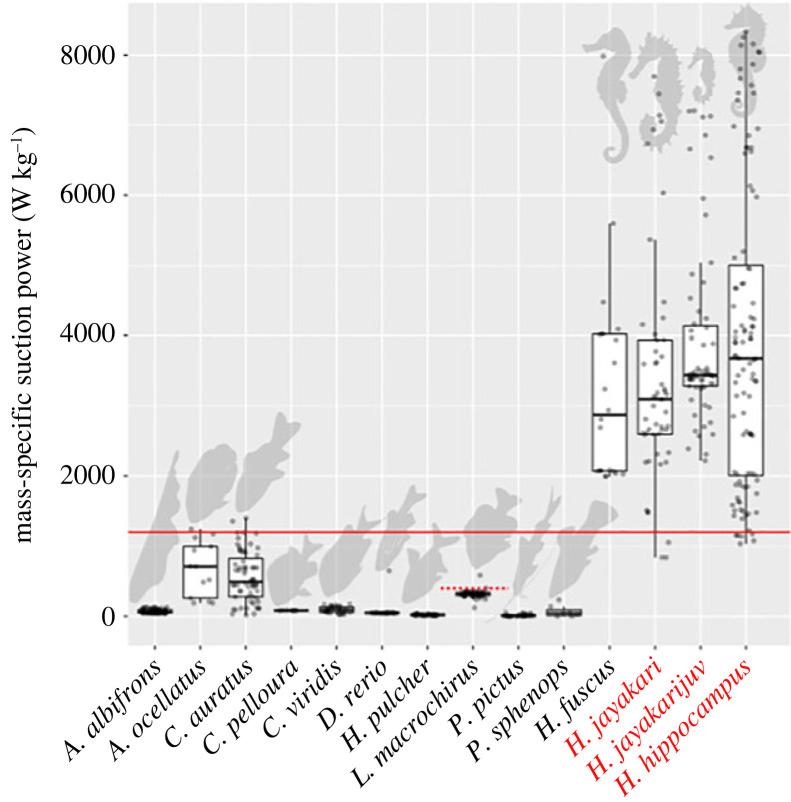
Mass-specific power of suction feeding for seahorses (*Hippocampus fuscus, H. jayakari and H. hippocampus*) is threefold higher than the maximum recorded from or any vertebrate muscle (1121 W kg^−1^; solid red line; [30,34]), indicating that seahorses use an LaMSA system to power their suction flows. A phylogenetically informed mixed-effect model indicated very strong support (Bayes factor > 10^5^) for the effect of LaMSA on mass-specific suction power (electronic supplementary material, table S3). Power for both LaMSA (red font) and non-LaMSA fishes (black font) was estimated by using the PIV-measured flow field to jointly estimate buccal pressure and suction volume (electronic supplementary material, equation S3) and hence refers to net suction power, i.e. the power used to accelerate the water outside the mouth. Boxes encompass the second to third quartile range, and the horizontal black line is the median estimated power for each species. The dashed red line indicates the estimated peak suction feeding power for *L. macrochirus* from [35], which agrees with the results from the method used in this study. We therefore conclude that the LaMSA system in seahorses has a dual function, powering both pivot feeding and suction feeding.

We hypothesized that the LaMSA system in seahorses is recruited to enhance suction flows and hasten their occurrence. One way of identifying an LaMSA is to compare the power required by the system with the power capacity of the muscles that drive the system [3]. We calculated the power used to accelerate the water into the mouth by integrating the suction pressure with respect to buccal volume over time (see the electronic supplementary material, ‘*Suction flow power*’ and equation S3; [29]). We used an established method to calculate the time-varying pressure fields from the particle imaging velocimetry (PIV) flow fields [30,31] to estimate the pressure at the mouth orifice (figure 2*b*; electronic supplementary material, figure S3). To estimate the instantaneous flow rate of water into the buccal cavity, we multiplied the instantaneous mouth diameter (observed in the high-speed videos) and the flow speed into it (estimated from the PIV). We integrated these estimates of suction pressure and flow rate to calculate the power used to accelerate the water outside of the mouth during a suction feeding event (hereafter ‘net suction power’; see the electronic supplementary material, ‘*Suction flow power*’ and equation S3). We note that the net suction power is lower than the total suction power because it does not account for the power required to accelerate the skeletal and muscle tissues that make up the buccal cavity. That residual power was about 4% of the total suction power in largemouth bass [13], but could possibly be higher in seahorses. Net suction power was calculated for a subset of approximately 260 PIV videos of 13 seahorses from the three species and was 1.63 ± 0.10, 1.56 ± 0.12, 3.76 ± 0.18 and 1.08 ± 0.04 W (mean ± s.e.) for adult *Hippocampus jayakari*, *Hippocampus fuscus*, *Hippocampus hippocampus* and *H. jayakari* juvenile, respectively (electronic supplementary material, figure S6). Average mass-specific suction power (defined as suction flow power divided by the mass of the epaxial and hypaxial muscles that connect to the system) was 3423 ± 223, 3266 ± 310, 4263 ± 207 and 3911 ± 154 W kg^−1^ for adult *H. jayakari*, *H. fuscus*, *H. hippocampus* and *H. jayakari* juvenile, respectively. This mass-specific net suction power is approximately threefold the maximum recorded from any vertebrate muscle (1121 W kg^−1^ [33,34]). We further calculated the suction flow power for 10 other non-LaMSA actinopterygian species (*n* = ∼340 PIV videos from 30 individuals; see the electronic supplementary material, table S1 for number of realizations for each species; and ‘*Flow power*’ for details on calculation) and found that seahorses use significantly more power during suction feeding than other actinopterygians (phylogenetically informed mixed-effect model; Bayes factor > 10^5^; see the electronic supplementary material, table S3 for model results and details on statistical model). The estimated mean mass-specific power (±s.e.) for non-LaMSA fishes was 129.0 ± 301.4 W kg^−1^, supporting the general lack of elastic energy storage in non-Syngnathiformes fishes (figure 3). Conversely, the non-overlapping estimate (±s.e.) for seahorses was 3455.1 ± 246.9 W kg^−1^. Thus, the average mass-specific suction power for the *Hippocampus* species was fivefold higher than that recorded for *Astronotus ocellatus,* the highest across other actinopterygians (674 ± 94 W kg^−1^), and approximately 10-fold the maximal value measured for bluegill, *L. macrochirus* (321 ± 18 W kg^−1^; this study as well as Camp *et al*. [35]; figure 3), a species regarded as a high-performance suction feeder. We therefore conclude that the LaMSA system in seahorses is used to power both pivot feeding and suction feeding, resulting in coordinated, extremely fast head rotation and buccal expansion.

### (b) Estimation of tendon power

**Figure 4.**
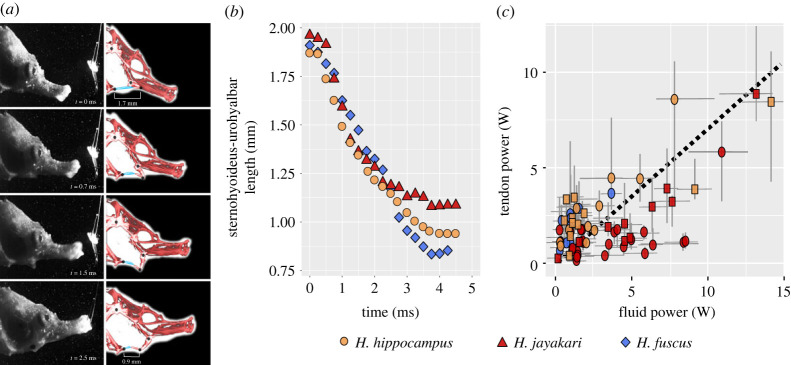
Recoil of the sternohyoideus tendons provides much of the suction power in seahorses, suggesting an additional source of elastic energy storage in addition to the previously recognized epaxial tendons. (*a*) Time series with images from high-speed video on the left and a digitally edited interpretation of the bones (red) and sternohyoideus tendons (blue) on the right. The joints of the four-bar lever system are represented by black points. (*b*) Digitizing the joints of the flexible bar (i.e. the edges of the hyoid and cleithrum) indicates that the sternohyoideus tendons shorten by an average of 48 ± 12% during these strikes (electronic supplementary material, movie S5). (*c*) Comparison of the net suction power estimated from PIV flow fields (‘fluid power’; *x*-axis in (*c*)) and the power contributed by the sternohyoideus tendons (‘tendon power’; *y*-axis in (*c*)) reveals that the tendons can provide, on average, approximately 72% of the net suction power. Power contributed by the compressing sternohyoideus tendons was estimated using the measured contraction of the tendons ((*b*); from the PIV movies) and the length-force curve of the tendons measured using a material testing apparatus (electronic supplementary material, equation S4). A phylogenetically informed mixed-effect model was used to estimate the slope between the power contributed by the recoiling sternohyoideus tendons and net suction power. The estimated slope ± s.e. was 0.72 ± 0.07 (*n* = 67 videos from five individuals of three species; electronic supplementary material, table S4). Colours represent different species and shapes represent the individuals within each species (see the electronic supplementary material, table S1). For error calculation see the last paragraph of the electronic supplementary material, '*Suction flow power'* and '*Tendon power'.*

In seahorses, head rotation is achieved through the recoil of the epaxial tendons, connecting to the supraoccipital bone at the back of the head (figure 1*a*; [36]). Previous studies have modelled the hyoid movement in pivot-feeding Syngnathiformes as part of a four-bar lever system that transmits force and movement from the epaxial tendons (figure 1*a*; figure 4*a*; [12,14,37]; ‘single-LaMSA system’ in the electronic supplementary material, movie S1). Buccal expansion is driven by hyoid retroversion, which depresses the floor of the mouth cavity and drives a lateral head expansion. According to this model, elastic energy is stored in the epaxial tendon, i.e. this LaMSA mechanism is powered by a single elastic element that powers head rotation [22]. Analysis of cleared and stained seahorse specimens revealed that one of the links (link no. 4 in figure 1*a*) is not rigid, because the hyoid is connected to the cleithrum by the sternohyoideus muscle-tendons complex (composed of the hypaxial and sternothyroid muscles and their associated tendons; see [22]; the electronic supplementary material section ‘*Morphology*’). In a rigid four-bar system, it is possible to calculate the movement of one link (i.e. the head) based on the movement of any other link (e.g. the hyoid) [15,38]. We could therefore test whether this link length is fixed by comparing the observed (digitized) movement of the hyoid and head to that expected for a rigid four-bar system ([15,38]; see the electronic supplementary material, ‘*Kinematics*’). The expected angle differs by 17 ± 6 degrees from the observed head elevation angle (*n* = 264 strikes and 13 individuals). We therefore assert that the transmission of motion and power within a seahorse's head is not consistent with a rigid four-bar system. Specifically, we suggest that the sternohyoideus tendons contract during suction feeding [22], potentially releasing elastic energy that contributes to the generation of suction flows. Indeed, tracking the two attachments of this flexible bar (the distal end of the hyoid and the ventral protrusion of the cleithrum) revealed that the entire bar compresses by an average of 48 ± 12% during hyoid retroversion (figure 4; see the electronic supplementary material, ‘*Kinematics*’, figure S5 and movie S5).

To test the power contributed by the sternohyoideus tendons, we dissected and measured the force-length relationships of the tendons in five individuals belonging to the three seahorse species (electronic supplementary material, table S1 and figure S5) and calculated the power transmitted from the compressing tendon to the system, based on the tendon recoil observed from the video used for the PIV (figure 4*c*; see the electronic supplementary material, ‘*Tendon power*’ and movie S5). The estimated power contributed by the sternohyoideus tendons was 5.02 ± 0.62, 3.30 ± 0.42 and 2.73 ± 0.40 W (mean ± s.e.) for adult *H. jayakari*, *H. fuscus* and *H. hippocampus*, respectively (juveniles were too small to dissect the tendons). Furthermore, there was a strong correlation between the power contributed by the compressing sternohyoideus tendons and the power required by the suction flow (phylogenetically informed mixed-effect model, Bayes factor > 10^5^; estimated slope ± s.e. = 0.72 ± 0.07; see the electronic supplementary material, table S4 for model results and details on statistical model). Altogether, these estimates indicate that the sternohyoideus tendons can contribute most of the power needed to accelerate the suction flows into the mouth by directly pulling on the hyoid. While our estimate of the power contributed by the tendons is lower than our estimated suction flow power (i.e. 72%), this is probably owing to the conservative nature of calculating tendon power (see the electronic supplementary material, '*Tendon power*'). Furthermore, the sternohyoideus tendon continues into the sternohyoideus and hypaxial muscles, potentially storing more energy than we accounted for in our material testing. It is also possible that the access power is contributed by the epaxial tendons through the four-bar lever system. Unfortunately, it is not possible to track the dynamics of the epaxial tendons from our videos, and we are not able to quantify its contribution.

## 3. Discussion

We conclude that the LaMSA system in seahorses represents a unique mechanism in which two masses are accelerated simultaneously by the same system (i.e. the head as it swings upwards and the water sucked into the mouth). The system is powered by both the epaxial and the sternohyoideus tendons, whose movements are coordinated, apparently by virtue of sharing the same latch system [39,40]. LaMSA systems in which two elastic elements are apparently rare in natural systems (mantis shrimp [18]; trap-jaw ants [41]) and the implications of this design are not fully understood. In mantis shrimp, this design might help trigger the system by allowing it to over-centre [25]. In trap-jaw ants, the design is thought to enable the hinge-less motion of the jaws [41]. Here we suggest that this design helps to coordinate the acceleration of head elevation and suction flow, which is necessary for prey capture. This coordination allows the fishes to draw their prey into the mouth before an escape response can be initiated and is especially important when feeding on copepods, which have myelinated axons enabling very rapid responses to hydrodynamic ques (within 4 ms) and escape accelerations of up to 300 m s^−2^ [42,43]. With head elevation and suction flow occurring sooner than the prey's escape latency, attacks can be successful even though the power amplification does not break constraints on spatial patterns: i.e. suction flows only affect a region of approximately 1 gape diameter away from the mouth. The fast flows, executed at the right timing, can overwhelm the prey before it can be alerted by the moving snout and the accelerating suction flows that are much briefer than the copepod's reaction time [44,45]. Delaying the suction flow until after the predator's mouth has arrived near the prey (i.e. without coordination) would have provided the copepods with time to escape in response to the wake generated by the moving snout.

The estimated power contributed by the tendons is lower than our estimated suction flow power (i.e. 72%). This could be accounted for by power transmitted from the epaxial tendon through the four-bar linkage system. However, it is possible that elastic energy is also stored within other structures. For example, elastic energy could be stored within the epaxial and hypaxial muscles, as muscles have been found to store elastic energy through the interaction of titin and actin [39,40]. Additionally, the long contraction phase to load the tendons could potentially increase muscle power output even at such high contraction velocities [46]. It is also conceivable that bone deformation within the cranium aids in this rapid elastic motion, although we did not detect changes in the lengths of the rigid elements of the four-bar system from our external landmarks.

In the sister families Centriscidae (snipefishes) and Fistulariidae (cornetfishes), the sternohyoideus tendon is ossified through most of its length to create the urohyal, forming a more rigid four-bar lever system [38]. The flexible tendons, while still present, are significantly reduced. Although the power amplification dyad in seahorses is probably the derived state within Syngnathidae, to the best of our knowledge a systematic investigation of urohyal ossification within Syngnathiformes has not been conducted. Pipefishes and snipefishes both use an LaMSA system to power pivot feeding [12,14] and the rapid movement of their hyoid implies that suction flows are coordinated with head elevation, similar to seahorses. This also implies that suction feeding in these ossified species is also power-amplified. However, it is unknown how suction flows in these species compare with non-amplified flows, or with seahorse's suction flows. Specifically, it was recently proposed that an elastic ligament within the four-bar linkage system of Stomatopods can serve as a latch, by allowing the reversal of the torque on one of the bars [25], and it is likely that the sternohyoideus tendons in seahorses can serve a similar function. The occurrence of such a mechanism in seahorses could drive a performance difference between basal Syngnathiformes with a more rigid four-bar system (‘single-spring LaMSA’ species) and those with elongated sternohyoideus tendons (‘dual-spring LaMSA’ species), because latch dynamics should strongly affect the output power in LaMSA systems [17,47]. The modifications of LaMSA mechanics in Syngnathiformes, together with their tractable evolution, makes this family a compelling case study of how the functional structure of LaMSA systems affect their evolutionary dynamics.

## Data Availability

The data is available at https://doi.org/10.5061/dryad.9zw3r22h6 [48]. Data are also provided in the electronic supplementary material [49].
